# Supplementary material for: Whole body periodic acceleration improves survival and microvascular leak in a murine endotoxin model
Source: PLoS One. 2019 Jan 25;14(1):e0208681. doi: 10.1371/journal.pone.0208681 (PMC6347233; doi:10.1371/journal.pone.0208681)
Supplement: S1 File — A table also contains Behavioral Scoring Criteria in Mice, amended to include stool quality criteria, a maximal worse score is 32. (PDF) [file pone.0208681.s001.pdf]

SUPPORTING INFORMATION

Materials & Methods

Protein Expression and Phosphorylation

As previously describe by our laboratory, the entire heart was minced and homogenized at 4 °C followed by one step total protein extraction with an Extraction Buffer System (Invitrogen Corporation, Carlsbad, CA)

The extracted cardiac protein was measured by the BCA Protein Assay (Thermo Fisher Scientific, Waltham, MA) on a SpectraMax Plate Reader (Molecular Devices, Sunnyvale, CA). Individual proteins were then analyzed by Western blot. Equal amounts of total protein were separated on 4-12% NuPAGE Novex Bis-Tris SDS-PAGE Gels (Invitrogen Corporation, Carlsbad, CA) and transferred to Immobilon-FL PVDF membrane (Millipore Corporation, Billlerica, MA). The PVDF membrane was treated with a blocking agent (GE Healthcare Bio-Sciences Corporation, Piscataway, NJ) and probed with primary, fluorescein-linked secondary antibodies as well as anti-fluorescein alkaline phosphatase conjugate. The following primary antibodies were used; TIE2 (24859, 1:2000), pTIE2 (192800, 1:2000), (Abcam, Cambridge, MA). Individual Protein loading controls used are: Glyceraldehyde-3-phosphate dehydrogenase (GAPDH 9545, 1:10000, Abcam, Cambridge, MA). Blots were visualized by Enhanced Chemifluorescence (ECF) (GE Healthcare Bio-Sciences Corporation, Piscataway, NJ) on Storm 860 Imaging System (GE Healthcare Bio-Sciences Corporation, Piscataway, NJ). The Storm 860 Imaging System exhibits a

linear response to fluorescent signal intensities and protein levels were quantified using ImageQuant software (GE Healthcare Bio-Sciences Corporation, Piscataway, NJ). [1-3].

Euthanasia

After completion of each of the experimental protocols or after reaching the humane endpoint of behavioral score of 28 , animals were euthanized by a dose of Ketamine 90mg/kg and Xylazine 25mg/kg, followed by pentobarbital 100mg/kg IP, until absence of corneal and pedal reflex, and no electrical activity on ECG, and decapitation via guillotine, a method approved by the American Veterinary Medical Association Guidelines on Euthanasia. Perfusion and or organ harvesting was performed as per protocol [4-6]

Animal Attrition In Survival Experiments

Animal Group	*n*	*Euthanized Prior to 48 hrs*
LPS-CONT	12	11
pGz-LPS	12	5
LPS-pGz	12	2
L-NAME- LPS-CONT	8	8
L-NAME- pGz-LPS	8	8
L-NAME- LPS-pGz	8	8

S1. Table A

Behavioral Scoring Criteria

The below criteria was modified from Shrum et al to include stool quality. The maximum score is 32.[7]

Variable	Score and description
Appearance	0- Coat is smooth
	1- Patches of hair piloerected
	2- Majority of back is piloerected
	3- Piloerection may or may not be present, mouse appears “puffy”
	4- Piloerection may or may not be present, mouse appears emaciated
Level of consciousness	0- Mouse is active
	1- Mouse is active but avoids standing upright
	2- Mouse activity is noticeably slowed. The mouse is still ambulant.
	3- Activity is impaired. Mouse only moves when provoked, movements have a tremor
	4- Activity severely impaired. Mouse remains stationary when provoked, with possible tremor
Activity	0- Normal amount of activity. Mouse is any of: eating, drinking,

	climbing, running, fighting
	1- Slightly suppressed activity. Mouse is moving around bottom of cage
	2- Suppressed activity. Mouse is stationary with occasional investigative movements
	3- No activity. Mouse is stationary
	4- No activity. Mouse experiencing tremors, particularly in the hind legs
Response to stimulus	0- Mouse responds immediately to auditory stimulus or touch
	1- Slow or no response to auditory stimulus; strong response to touch (moves to escape)
	2- No response to auditory stimulus; moderate response to touch (moves a few steps)
	3- No response to auditory stimulus; mild response to touch (no locomotion)
	4- No response to auditory stimulus. Little or no response to touch. Cannot right itself if pushed over
Eyes	0- Open
	1- Eyes not fully open, possibly with secretions
	2- Eyes at least half closed, possibly with secretions
	3- Eyes half closed or more, possibly with secretions

	4- Eyes closed or milky
Respiration rate	0- Normal, rapid mouse respiration
	1- Slightly decreased respiration (rate not quantifiable by eye)
	2- Moderately reduced respiration (rate at the upper range of quantifying by eye)
	3- Severely reduced respiration (rate easily countable by eye, 0.5 s between breaths)
	4- Extremely reduced respiration (>1 s between breaths)
Respiration quality	0- Normal
	1- Brief periods of labored breathing
	2- Labored, no gasping
	3- Labored with intermittent gasps
	4- Gasping
Stool	0- Normal
	1- Frequent or a lot
	2- Loose
	3- Diarrhea
	4- No stool
TOTAL	Score
	**MAX WORSE SCORE 32**

REFERENCES

1. Wu H, Jin Y, Arias J, Bassuk J, Uryash A, Kurlansky P, et al. In vivo upregulation of nitric oxide synthases in healthy rats. Nitric Oxide. 2009;21(1):63-8. doi: 10.1016/j.niox.2009.05.004. PubMed PMID: 19481168; PubMed Central PMCID: PMCPMC3135669.

2. Wu H, Uryash A, Bassuk J, Kurlansky P, Giridharan GA, Shakeri M, et al. Mechanisms of Periodic Acceleration Induced Endothelial Nitric Oxide Synthase (eNOS) Expression and Upregulation Using an In Vitro Human Aortic Endothelial Cell Model. Cardiovascular Engineering and Technology. 2012;3(3):292-301. doi: 10.1007/s13239-012-0096-4.

3. Uryash A, Wu H, Bassuk J, Kurlansky P, Sackner MA, Adams JA. Low-amplitude pulses to the circulation through periodic acceleration induces endothelial-dependent vasodilatation. J Appl Physiol (1985). 2009;106(6):1840-7. Epub 2009/03/28. doi: 10.1152/japplphysiol.91612.2008. PubMed PMID: 19325024.

4. Nemzek JA, Xiao HY, Minard AE, Bolgos GL, Remick DG. Humane endpoints in shock research. Shock. 2004;21(1):17-25. doi: 10.1097/01.shk.0000101667.49265.fd. PubMed PMID: 14676679.

5. Toth LA. Defining the Moribund Condition as an Experimental Endpoint for Animal Research. ILAR journal / National Research Council, Institute of Laboratory Animal Resources. 2000;41(2):72-9.

6. Morton DB. A systematic approach for establishing humane endpoints. ILAR journal / National Research Council, Institute of Laboratory Animal Resources. 2000;41(2):80-6. PubMed PMID: 11406701.

7. Shrum B, Anantha RV, Xu SX, Donnelly M, Haeryfar SM, McCormick JK, et al. A robust scoring system to evaluate sepsis severity in an animal model. BMC research notes. 2014;7:233. doi: 10.1186/1756-0500-7-233. PubMed PMID: 24725742; PubMed Central PMCID: PMC4022086.
